# Mobility, ICT, and health: a built environment investigation of older Chinese migrants’ social isolation and loneliness

**DOI:** 10.1186/s12889-025-21750-3

**Published:** 2025-02-07

**Authors:** Amber DeJohn, Bochu Liu, Xinlin Ma, Michael J. Widener, Zhilin Liu

**Affiliations:** 1 Department of Geography, Florida State University, Tallahassee, FL USA; 2 College of Architecture and Urban Planning, Tongji University, Shanghai, China; 3 Department of City and Regional Planning, University of North Carolina at Chapel Hill, Chapel Hill, NC USA; 4 Department of Geography & Planning, University of Toronto, Toronto, ON Canada; 5 School of Public Policy & Management, Tsinghua University, Beijing, China

**Keywords:** Social isolation, Loneliness, Mobility, ICT, Older adults, Migrants, Mental health, Physical Health

## Abstract

**Background:**

Social isolation and loneliness have detrimental impacts on health, especially for older adults. During the COVID-19 pandemic, physical access to third places (e.g., coffee shops, libraries) decreased due to the closure of non-essential destinations and personal risk assessments. Older adults reported adopting information and communication technology (ICT) during pandemic lockdowns. ICT-mediated socializing may have different impacts on loneliness than in-person equivalents. Understanding access to social connection and their distinct relationships to the built environment and health for older Chinese migrants is critical to supporting equitable, healthy aging in a post-COVID world.

**Methods:**

Using a survey of older Chinese migrants in the Greater Toronto Area (GTA) during the extended COVID-19 lockdown, we investigate how community mobility and ICT use, two mechanisms of socializing, relate to the built environment and influence loneliness (De Jong Gierveld 6-item scale), as well as mental and physical health (SF-12). Specifically, we use a structural equation model to test a theoretical framework of older adult social isolation.

**Results:**

Our model demonstrates the importance of community mobility for reducing feelings of loneliness, while ICT use is significantly related to better physical health. Both community mobility and ICT use have significant, although opposite, relationships to transit density.

**Conclusions:**

Results indicate that ICT use might have limited ability to reduce loneliness and support mental health when mobility is limited. Addressing older migrants’ barriers to community mobility is critical to reducing feelings of loneliness.

**Supplementary Information:**

The online version contains supplementary material available at 10.1186/s12889-025-21750-3.

## Introduction

A substantial literature is developing concerning the activities and well-being of older adults during the COVID-19 pandemic [1–4], which is adding to our broader understanding of how older adults socialize when their mobility is limited. Many studies of older adults during COVID-19 originate from a variety of Western countries, including the UK, Netherlands, and the US. In general, these studies focus on a general older adult population [5, 6] or subgroups of suspected vulnerability, like older adults who live alone [2] or have health concerns [7, 8]. Relatively less work has focused on older migrant populations, specifically those who were facing new mobility barriers as they were unable to travel to see family and friends in their home country [9]. In addition, Asian migrants may have experienced worsened social conditions in their receiving countries that could have impacted their sense of belonging [10].

The Greater Toronto Area (GTA) is the largest metropolitan area in Canada and home to a considerably large Chinese community [11], which makes it a key region to evaluate the social contexts of older Chinese migrants. Not only is Toronto home to a substantial Chinese migrant community, but it also experienced extended COVID-19-related lockdown policies that limited access to destinations where older Chinese migrants socialize (e.g., culturally specific restaurants). Essential destinations that remained open, like grocery stores, represented the majority of locations of anti-Asian hate crimes during COVID [10]. This could have created degraded or uncertain social contexts for older Chinese adults, and their community perceptions may have been influenced. This social context positions Toronto as a critical area for studying social isolation during the pandemic.

An individual’s perception of their physical and social environment are important for understanding their physical and emotional health [12, 13]. Asking how older Chinese migrants in the GTA were able to access social destinations (physical or virtual) and the relationship between these socializing modalities, the built environment, and health during the pandemic will lend insight into the relationship between information and communication technology (ICT) use and place in times when physical environments are potentially less accessible. The first objective of this study is to identify how the built environment contextualizes older Chinese migrants’ perception of access to socializing mechanisms (mobility or ICT) during COVID-19 lockdown. We argue that social isolation is most pronounced when an individual has limited mobility and deficient access to ICT. Then, we seek to elucidate how mobility or ICT access influence loneliness and health.

Social isolation is detrimental to an individual’s health [14, 15], with one study finding that social isolation is more predictive of mortality than smoking [16]. Social isolation can be objective (e.g., the number of social interactions an individual has in a given time period) or perceived (e.g., the individual perceives a deficiency in their social network) [17]. Perceived social isolation (hereafter, loneliness) affects physical and mental health in a myriad of ways [18, 19]. Not only does social isolation degrade an individual’s sleep [20], mental health [21], and physical health [22], but it also advances cognitive aging [23]. Understanding how to support social connection and reduce isolation is key to bolstering health among vulnerable migrant populations.

When considering migrants versus native-born populations, differing social networks emerge that have unique relationships to health and built environments [24, 25]. For example, Fokkema and Naderi (2013) investigated loneliness specifically among an older Turkish migrant group and the native-born population in Germany, finding that migrants experience higher levels of loneliness. However, when controlling for socioeconomic status and health, this relationship was no longer statistically significant. In a study of older Chinese internal migrants’ social capital, bonding social capital (e.g., relationships with people from your own community) is shown to be more important for both migrants and native-born older adults’ mental health, while bridging and linking social capital (e.g., relationships with people from different communities) is only important for older migrants’ mental health [25]. This suggests that migrant populations who are isolated from native-born or culturally different individuals may experience worsened mental health. Tang et al.’s (2020) study found that access to public transportation is related to improved mental health for the migrant and native-born older Chinese populations, perhaps because of transit’s ability to increase access to destinations among a population that relies on it more than those in the West [25].

Built environments can facilitate or limit access to destinations where socializing occurs. In a car-dependent built environment, having access to a personal vehicle may bolster travel to social destinations [26], while age-related mobility disabilities or lost access to a personal vehicle are potential barriers [27, 28]. Public transit [29] and individual mobility (e.g., walking or using a mobility aid) are key to accessing socializing destinations [30–32]. Pandemic-era literature in the Toronto-context has demonstrated the quick return of older adults to using public transit [33], which indicates that older adults have lower demand elasticity for public transit modes.

Elements of the built environment beyond the transportation landscape are also important for older adults’ mobility, including a sense of safety from crime, age-friendly walking facilities (e.g., even pavements, benches), and the presence of social destinations [32, 34]. Community mobility is also shaped by the individual’s self and social identity, which impacts an older adult’s perception of mobility (e.g., social stigma due to a mobility aid) [30]. This is especially important in the context of older Chinese migrants during the COVID-19 pandemic, who may have developed new perceptions of their community in addition to changing attitudes about public transportation [35]. But beyond physical communities and the individuals’ perception of them, virtual spaces exist as a complement.

During the COVID-19 pandemic, older adults were widely adopting new information and communication technology (ICT) or using ICT more frequently due to a loss of in-person socializing destinations [6, 8, 36, 37]. Before the pandemic, migrants were already using ICT to maintain social relationships with family in their countries of origin [38]. While older adults may experience different barriers to adopting ICT than the general adult population, including physical or cognitive impairment or anxiety [39], ICT adoption rates amongst older adults were generally increasing before the pandemic began [40]. This may indicate a sustained spike in ICT adoption rates into the future. Given changes in how this population engages in socializing, it is important to interrogate how ICT may serve as an avenue for reducing social isolation among older adults who may face mobility barriers and difficulties socializing in person [41, 42].

**Fig. 1:**
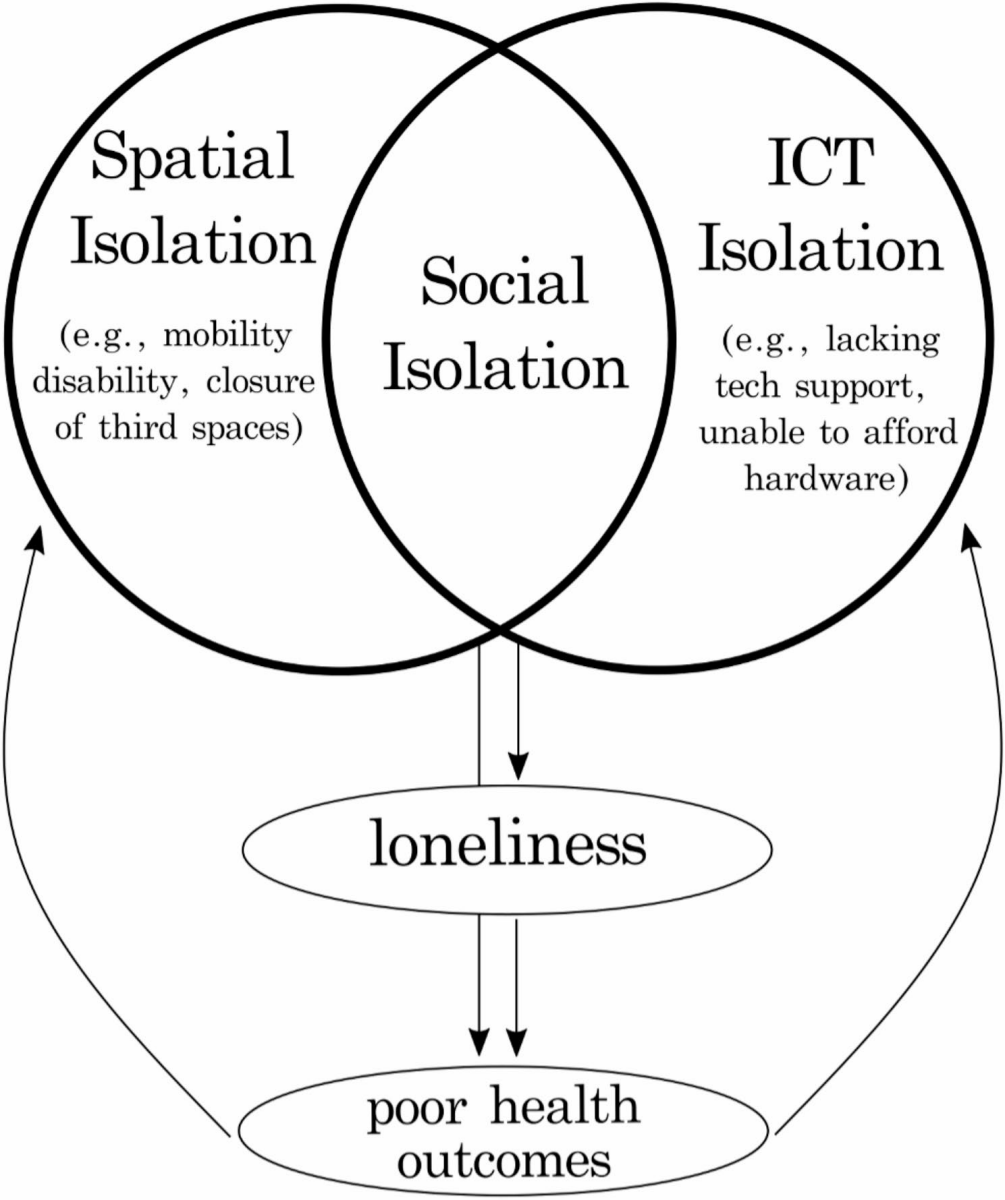
Graphical representation of theoretical framework developed from literature review, which guides the hypothetical model used during modeling

To summarize this brief literature review, social isolation and loneliness have well-established links to poor health outcomes and are heavily dependent on the individual’s ability to enact their social agency within their individual context. Mobility, both within their immediate city or across international borders, could promote their ability to connect. While ICT seemingly boasts clear advantages for older adults, the scale of these benefits is still unclear. By bringing together issues of mobility and ICT use, we might learn how to better support healthy aging among an older migrant community. Figure 1 provides a simple graphical rendering of how spatial and ICT isolation interact to produce social isolation, which can directly impact feelings of loneliness and poor health outcomes. The COVID-19 pandemic, a time when people were spatially isolated, presents an opportunity to study this phenomenon. This study does so by focusing on how older Chinese migrants in Toronto might have maintained social connections during Toronto’s extended lockdown.

Due to the importance of ICT socializing as a coping mechanism for spatial isolation, we hypothesize that loneliness will be dependent on both spatial and technological isolation. More specifically, we pose a series of hypotheses to test the relationship between spatial isolation, operationalized as community mobility, and technological isolation, operationalized as ICT use, and loneliness. First, we hypothesize that built environment characteristics will have an impact on both community mobility and ICT use. Second, we hypothesize that both ICT use and community mobility will have a direct effect on loneliness scores. Third, we hypothesize a direct and indirect effect of ICT use and community mobility on both mental and physical health, with the indirect effect mediated by loneliness. Ultimately, by testing these hypotheses, we aim to contribute to a comprehensive understanding of social isolation that is informed by an individual’s built context and feelings of belonging in a non-native country.

## Methods

### Survey

We conducted our survey of older Chinese migrants (60 years or older) who live in Toronto or surrounding municipalities (e.g., Richmond Hill, Mississauga) from March 2021 to June 2021. The geographic scope allowed us to recruit participants living in areas with high concentrations of Chinese migrants that are located outside the city, while also including a variety of built environments. To accommodate physical distancing and ensure the safety of participants during the extended COVID-19 lockdown, we recruited via physical flyers placed in two of Toronto’s Chinatowns, as well as virtual means such as the instant messaging app WeChat (community members sent recruitment messages in group chats), and paid advertisements on home pages of popular Chinese Canadian websites. We also allowed for referrals from study participants. To participate in the survey, individuals had to be 60 or older, speak Mandarin, and have immigrated to Canada from China. The language criterion was intended to make the sample comparable to a parallel sample collected in Beijing. Mandarin-speaking research assistants administered the survey over the phone. The main survey collected information about the individual’s migration information, household characteristics, mobility and community perceptions, health (SF-12) [43, 44], loneliness (De Jong Gierveld 6-item scale [45, 46], and ICT use. Once the main survey was complete, research assistants guided the participant through a single-day activity diary (see [47] for additional activity diary information). Participants were only surveyed Tuesday through Saturday to ensure the activity diary reference day (i.e., the day prior) was a weekday. We obtained ethical approval from the University of Toronto Research Ethics Board before data collection began.

### Participant characteristics

Our sample includes 96 Chinese immigrants. The median age is 70, and 64% are women. The median time in Canada (i.e., time since arrival) is 15 years. The majority (81%) have a spouse, at least one child living in the Toronto area (85%), and moved to Canada because they are dependent on adult children (66%). Most of our participants live with family members or non-relatives (85%). Living arrangements are quite complex, including extended family, immediate family, or friends. A table of all household arrangements can be found in Appendix 1. Approximately 68% of participants report it is somewhat or extremely easy to make ends meet financially, compared to 16% for whom it is somewhat or extremely difficult to make ends meet. The full participant characteristics are provided in Table 1. A map of participant home locations is available in Appendix 2.

**Table 1:** Older Chinese migrants sample characteristics (*n* = 96)

Variable	Sample
Age (years), mean, sd	70.29, 6.78
Years in Canada, mean, sd	15.86, 8.85
Gender, n (%)	
Woman	61 (63.54)
Man	35 (36.45)
Employment status, n (%)	
Working	15 (15.63)
Retired	79 (82.29)
Looking for work	0
Lost employment (COVID)	1 (1.04)
Working less (COVID)	1 (1.04)
Education, n (%)	
Elementary school and below	1 (1.04)
Junior high school	8 (8.33)
High school^a^	16 (16.67)
College/university	19 (19.79)
Bachelor’s degree	38 (39.58)
Above bachelor’s degree	14 (14.58)
Reason for immigration	
Dependent on adult children, relatives, etc.	63 (65.62)
Work	13 (13.54)
Take care of grandchildren	3 (3.13)
Other^b^	17 (17.71)
Living arrangement, n (%)	
Household members^c^	82 (85.42)
Living alone	14 (14.58)
Marital status, n (%)	
Spouse or long-term partner	76 (84.44)
Single or widowed	18 (15.56)
Financial difficulty, n (%)	
Extremely difficult	2 (2.08)
Somewhat difficult	13 (13.54)
Neither difficult nor easy	16 (16.67)
Somewhat easy	49 (51.04)
Extremely easy	16 (16.67)

^a^Includes technical secondary school and vocational high school

^b^Examples include family reunification, study, etc.

^c^Includes many arrangements (e.g., with spouse and non-relative, with children, grandchildren, and relative)

### Measures

To measure loneliness, we use the De Jong Gierveld 6-item scale [45], which is comprised of six questions about emotional and social loneliness, and from which we can derive a loneliness score ranging from zero (not lonely) to six (intensely lonely). Emotional loneliness occurs when an individual perceives a deficit in intimate relationships, while social loneliness indicates an individual feels a deficit in their wider social network [45].

We use the validated SF-12 questionnaire [43, 44] to measure participants’ self-rated health. It includes two sub-scores, one estimating mental health and the other estimating physical health. We calculate the sub-scores using publicly available weights in Python.

We calculate walkability scores using the proximity measures database available from Statistics Canada [48]. Walkability scores are built by summing proximity scores to childcare, employment, pharmacies, healthcare providers, grocers, educational institutions, libraries, parks, and transit facilities, then dividing by the maximum aggregate proximity score. We further simplify this variable by assigning each participant a quartile based on their walkability score. We also use dissemination area population density from the 2021 Canadian census to act as a signal for other built environment variables (e.g., walking facilities, retail density, etc.)

We include information about the transit environment by using GTFS files from various transit agencies in the Greater Toronto Area (GTA), including those in Barrie, Brampton, Burlington, Durham, Guelph, Hamilton, Kitchener-Waterloo, Milton, Mississauga, Niagara, Oakville, Toronto, and York, and the regional GO commuter rail. Specifically, we calculate the number of transit trips per hour that serve bus stops in the dissemination area normalized for the area served (i.e., the density of stops). These data and the code used to generate them are publicly available [49].

To measure perceived mobility, we employ Likert-scale survey questions for perception of community trust, inclusion, feeling the community attaches importance to the elderly, safety, ease of accessing a personal vehicle. For ICT use, we utilize survey questions about the participant’s comfort using ICT, time spent using technology (derived from the activity diary), perceived ability to use ICT when lonely, and frequency of social technology use before the pandemic.

### Structural equation model

To examine the influence of built environments, ICT use, and social isolation on the emotional health of older Chinese migrants, we use a structural equation modeling approach. The benefits of structural equation modeling is the ability to estimate latent variables based on a series of correlated questions (e.g., responses on ICT use behaviors) and to structure the explanatory variables in such a way that the pathways of relationships can be parameterized, enabling us to quantify direct and indirect effects. To construct our model, we follow the protocol provided in Kenneth Bollen’s [50] text: specification, identification, estimation, evaluation, re-specification, and interpretation.

**Fig. 2:**
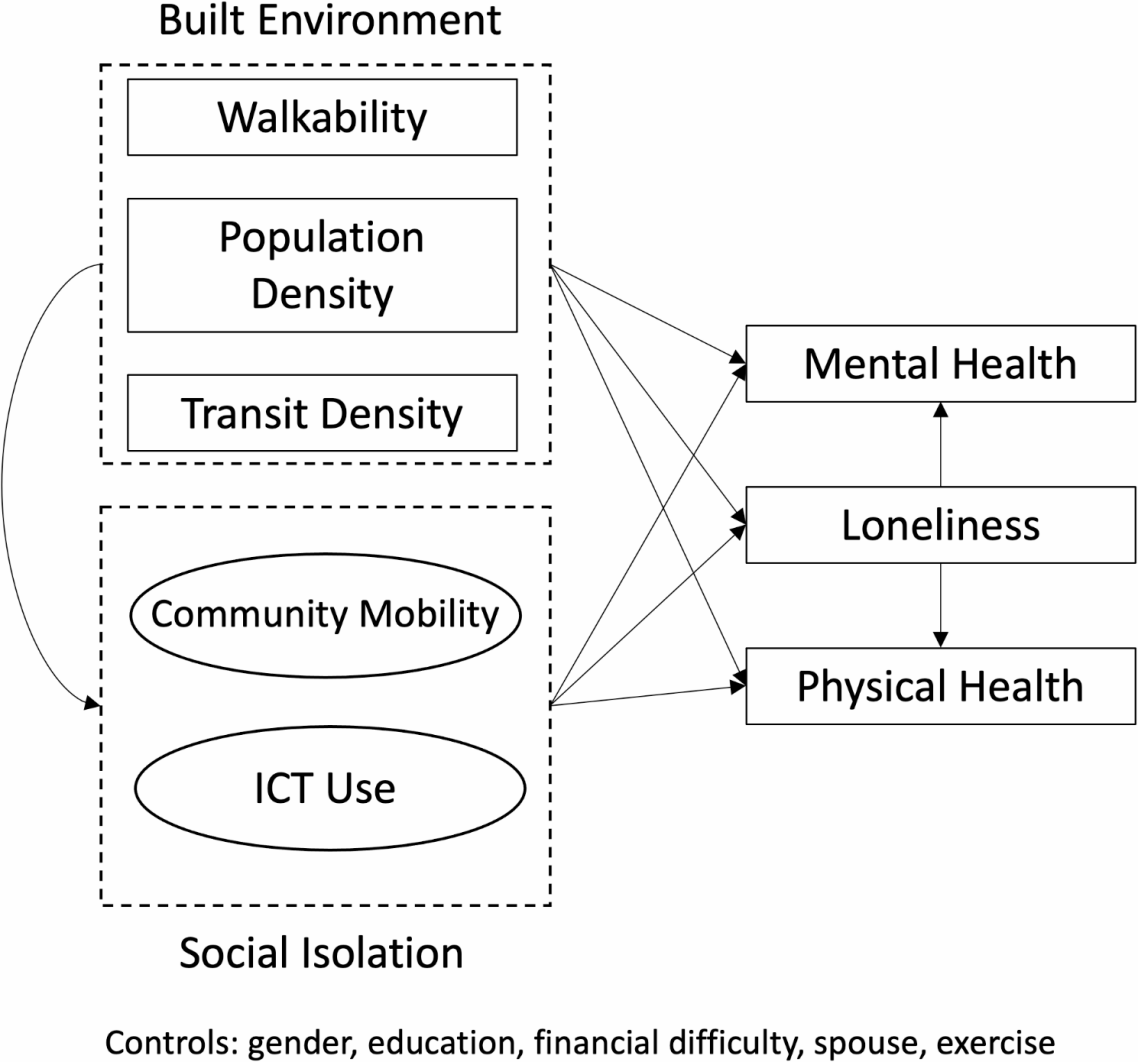
Conceptual diagram of structural equation model

During the specification and identification step, we build a conceptual model based on existing literature and determine if we have sufficient information to estimate the parameters. This conceptual model demonstrates the hypotheses and structure we wish to parameterize. Figure 2 provides a graphical representation of the conceptual model. While Fig. 1 presents the full framework based on the literature, Fig. 2 has some notable modifications that suit the small sample size (e.g., some reciprocal relationships are removed). In our conceptual model, we hypothesize there are two dimensions of social isolation during the lockdown: spatial and technological. We operationalize spatial isolation as a latent ‘community mobility’ variable, constructed from manifest built environment and community perception variables. We suspect community mobility will have direct and indirect relationships with mental and physical health. The indirect effect of community mobility is mediated by loneliness, but it also has a direct relationship. We operationalize the technological isolation dimension as ‘ICT use.’ We select key survey variables to denote ICT use: time using ICT and comfort using ICT. Latent variable indicators are provided in Table 2. Theoretically, we would expect there to be an interaction between community mobility and ICT use. We have not included this in the structural model to reduce complexity and promote model convergence. A correlation assessment revealed that the community mobility and ICT use latent variables are correlated (*r* = -0.21, *p* < 0.05). It should be emphasized that due to the small sample size, we must exercise restraint in adding variables to the model. Excessive variables result in misleading results (i.e., overfit) or failure to converge [51]. During the model identification step, we adjusted variable inclusion to ensure the model would run as expected (e.g., avoiding just-identified latent variables).

**Table 2:** Indicators used to construct latent variables

Construct	Indicator	Survey Question
Community mobility	Trust	People in my community can be trusted
	Inclusion	This community is very inclusive to residents of different backgrounds
	Elder importance	This community attaches great importance to the elderly
	Safety	My community is safe
	Travel convenience	It’s very convenient for residents living in this community to travel to other places
	Access to a personal vehicle	How easy is it for you to drive yourself in a private car
	Ease of walking	How easy is it for you to walk as a mode of transportation
ICT use	Comfort using ICT	I enjoy using technology to communicate with friends and family
	ICT social agency	When or if I feel lonely, I’m able to use technology to connect with friends and family with relative ease
	Pre-pandemic ICT use	How often did you connect with your friends and family through text messaging, phone calls, or WeChat before the pandemic
	Time spent using ICT	*Derived from the activity diary

We use a Diagonally Weighted Least Squares (DWLS) estimator. While the most used estimator is Maximum Likelihood, it assumes the data are continuous and have a multivariate normal distribution. Our data include an ordinal dependent variable (De Jong Gierveld 6-item loneliness score). DWLS is also preferred when the sample is relatively small, and the normality assumption is violated. We use R-package lavaan (version 0.6–12) to estimate our model. We evaluate our model’s overall fit (chi-square), relative fit (CFI, TLI), and absolute fit (Root Mean Squared Error of Approximation, Weighted Root Mean Residual). We assess indirect relationships using a bootstrapping method to generate standard errors and *p*-values. Our ultimate model and interpretation are provided in the results section.

## Results

In this section, we present the results of the structural equation model to understand the relationship between latent variables, the built environment, and health. Information about model fit is available in Appendix 3.

### Structural equation model results

Table 3 provides the standardized factor loadings (coefficients) for each latent construct. All factor loading estimates are statistically significant (*p* < 0.001) and greater than 0.30, a commonly used threshold denoting a moderate effect [52].

**Table 3:** Standardized factor loadings (coefficients) for latent constructs

Construct	Indicator	Standardized Estimate*
Community mobility	Trust	0.604
	Inclusion	0.697
	Elder importance	0.637
	Safety	0.511
	Travel convenience	0.534
	Access to a personal vehicle	-0.444
	Ease of walking	0.518
ICT use	Comfort using ICT	0.891
	ICT social agency	0.688
	Pre-pandemic ICT use	-0.604
	Time spent using ICT	-0.572

*All significant at the 0.001 level

These latent constructs built in the first part of the model are then used as explanatory variables for manifest (or observed) variables of loneliness, mental health, and physical health. Here, we included additional controls that are established correlates of each dependent variables.^1^ The results of the three regressions are available in Table 4. We assessed indirect relationships, but no statistically significant indirect effects were identified.

The community mobility latent variable has significant direct relationships to transit density, financial difficulty, gender, and education. Specifically, our model shows that as transit density increases, the perception of community mobility increases. We find a negative relationship between community mobility and financial difficulty and education; in other words, as financial difficulty and education level increase, perceived community mobility decreases. Identifying as a woman is also related to a higher perception of community mobility.

The ICT use latent variable has several significant direct effects. First, as transit density increases, ICT use decreases. Considering the ICT use construct was built using how much time an individual spent using ICT and their perceived ability to use ICT, this result may stem from individuals with greater access to public transit not relying on this method of socializing due to greater community mobility. We also observe that being a woman and having a spouse is related to lower ICT use.

**Table 4:** Structural equation model regression results

		Dependent Variables				
		**Community mobility**	**ICT use**	**Loneliness**	**Mental health**	**Physical health**
*Explanatory Variables*	**Constructs and mediators**:					
	Community mobility			-0.300**	0.079	-0.120
	ICT use			-0.059	-0.060	0.310**
	Loneliness				-0.538**	-0.152
	**Built environment**:					
	Walkability	0.089	-0.129	0.078	-0.176	0.120
	Population density	0.109	0.066	-0.031	0.172	-0.056
	Transit density	0.255**	-0.277**	-0.076	0.019	-0.003
	**Controls**:					
	Financial difficulty	-0.207**	-0.038	-0.096	-0.057	0.108
	Gender: Woman	0.167*	-0.340**	-0.014	-0.089	0.035
	Education	-0.255**	-0.166	0.051	0.096	0.115
	Spouse	0.129	-0.178*	-0.215*	0.022	0.164
	Pre-pandemic exercise					0.344**

***<0.01, ** <0.05, * < 0.10

We find several significant relationships to our primary outcomes of interest: loneliness, mental health, and physical health. First, we observed a significant and direct relationship between community mobility and loneliness. Individuals with better perceptions of community mobility have lower feelings of loneliness. Using loneliness as an explanatory variable for mental health, we find that as loneliness scores increase, mental health scores decline. We did not observe an indirect effect between community mobility and mental health, nor were there any other significant associations with mental health in our model. Finally, we observed a significant direct relationship between ICT use and physical health wherein an increase in ICT use is related to higher or better physical health scores. We see this relationship while controlling for how frequently a participant exercised before the pandemic, which itself also has a significant relationship to physical health scores.

## Discussion

This study is the first of its kind to investigate how older Chinese migrants’ perception of community mobility and ICT use relate to the built environment and emotional, mental, and physical health outcomes. The goal of this study was to evaluate what mechanisms might exist for older Chinese migrants to socially connect during a time when destinations might have been difficult to access due to COVID-19 policies or degraded social context. We focus on key factors that impact an individual’s ability to be spatially connected (i.e., community mobility) and connected via ICT (i.e., ICT use). The results of our structural equation model indicate that higher degrees of community mobility relate to reduced levels of loneliness during the extended COVID-19 lockdown, while higher ICT use was related to better physical health.

We first hypothesized that community mobility and ICT use would be dependent on built environment characteristics. Our model indicates that higher transit density was related to higher community mobility. Community mobility includes community perception factors, which suggests transit-rich residential contexts may have higher perceived trust, inclusion, safety, and elder importance. Put simply, positive community perceptions are entangled with greater spatial agency. Older adults’ built context can provide access to socializing destinations, with public transportation offers one key avenue for reducing social isolation in later life [29]. While our study focuses on an urban area where there is generally robust public transport, most of our participants report driving as their primary mode. This may be because our sample primarily lives in areas with a relatively lower population density. In Toronto, there exist pockets of lower transit access where individuals, especially those who are more socially vulnerable like migrants, may struggle to access destinations [53]. These areas may be additionally burdened with weaker sense of community.

Interestingly, our results suggest that higher transit density is related to lower ICT use. This is possibly due to older migrants in more transit-rich environments being more mobile in general, rendering them less reliant on the use of ICT to socialize. Pandemic-era literature shows that older adults are likely to remain transit riders in Canadian cities [33, 35], and this might be especially true for those who are not able to easily walk to destinations [54]. Given older Chinese migrants in relatively transit-poor areas have higher ICT use, these populations may be strategic for interventions that implement new technologies for accessing mobility services [55] as they are already exhibiting some level of comfort using ICT. It is also possible, although not explored here, that migrants with higher ICT use may be more embedded in physically distant social networks that require ICT for maintenance.

Before estimating our model, we hypothesized a direct relationship between community mobility and ICT use on loneliness scores (hypothesis 2). We observed that as community mobility increases, loneliness scores improve, indicating individuals with better community mobility are less lonely. However, we find no relationship between ICT use and loneliness. As the community mobility latent variable is built using perceived community safety and inclusion in addition to perceived mobility, it is possible that individuals who live in communities with higher proportions of migrants, which is typical of our sample, feel safer and better able to socially connect. In the greater context of increased anti-Asian sentiment, this result suggests that individuals embedded in immigrant social enclaves experience higher community mobility and enjoy access to their social network. Chinese migrants tend to have high levels of bonding social capital (e.g., social capital with individuals of the same class and background) [25]. Tang et al. (2020) suggested that linking or bridging social capital is more important for migrant mental health, yet we find no direct relationship between community mobility and mental health. This may reflect that our community mobility variable oversimplifies the types of communities and social capital to which a participant has access, and from which they derive emotional benefit. We also suspect our operationalization of community mobility may solely reflect bonding social capital, as our participants predominately lived in immigrant-rich social contexts and associated with other Chinese immigrants.

Our final hypothesis stated that ICT use and community mobility would have a direct and indirect effect on both mental and physical health. Our model results indicate that ICT use is directly related to improved physical health. We observed no direct or indirect effects of community mobility on mental or physical health. However, the finding that ICT is related to better physical health scores is worth noting. We observed this relationship despite controlling for pre-pandemic exercise, which was also significantly related to better physical health. This might contradict the common assumption that using ICT means an individual is somehow less physically healthy than others. Technological interventions have been widely successful at helping older adults exercise or physically rehabilitate [56–59], and older adults might have been particularly interested in using ICT to maintain physical activity during the pandemic [37]. While our ICT latent variable does not relate to a specific type of ICT or use case, it poses an interesting consideration for what might explain physical health or who might be an ICT user. Specifically, existing older adult ICT users may already leverage technology to maintain or promote physical health. Satisfactory physical health could be instrumental for improving access to in-person socializing opportunities [47], which our findings suggest can reduce loneliness. In other words, physical health may mediate the relationship between ICT use and loneliness.

Our results have implications for interventions aimed at supporting older adult socializing, mental, and physical health. First, alternative mobility options aimed at supporting individuals in car-dominated areas should be pursued. While most of our sample reported using a personal vehicle as their main mode of transportation, our model indicates that transit density is associated with higher community mobility. Elements of our community mobility variable that are difficult to address, like feelings of safety or trust, warrant further investigation. However, we suggest that community mobility may not have been impacted by increased anti-Asian sentiment since many of our participants lived in immigrant-dense residential areas and mainly associated with other Chinese immigrants. Our results also indicate that technological interventions may be best suited for targeting physical health outcomes. While there have been a number of studies showing the impact of ICT use on social isolation [41, 42], our model notably does not support this. This is potentially due to the inclusion of community mobility as a control or, as noted by Khosravi et al. (2016), the way we conceptualized and measured social connection. Future studies of technological interventions should include robust considerations and conceptualizations of socializing contexts, mobility, and how ICT encourages socializing beyond technological platforms.

## Limitations

Our study has some limitations that are worth noting. First, we recruited most of our sample using the social media platform WeChat or via referrals. This may have biased our sample toward older Chinese migrants who are already ICT users and socially connected. It should also be noted that the sample is relatively young, which poses another bias as the oldest older adults (i.e., 80+) have less ICT use compared to younger older adults [60]. A dissection by age might have revealed different patterns of ICT use and relationships to health. Additionally, our sample is small. Structural equation models are computationally complex and generally require large samples in order to converge. We limited the scope (i.e., number of variables) and complexity (i.e., types and amount of direct and indirect relationships) of our model to account for the small sample, but this remains a weakness of the analysis. Ideally, we would be able to collect a larger sample in the future so we can confirm these results, as well as investigate the link between community mobility and ICT use. We observed these variables are negatively correlated, but future work should identify reciprocal relationships both between these two variables and with health, which we have treated as only an outcome. Further, socializing context is lacking in our current model. A potential improvement would be including information about caregivers: whether the older adults are providing care to grandchildren or receiving care. This would paint a more complete picture of their daily lives and social interactions that might provide emotional benefit [61]. Additionally, our analysis lacks nuanced information related to safety and the experiences of an older migrant group. Recent literature has pointed to the limited emotional benefits of ICT during lockdown [37], but a sustained focus on older migrants ICT use and experiences of discrimination could advance our knowledge of migrant social enclaves [62].

In the same vein, we have used straightforward renderings of the built environment in our analysis. This was due to the small sample size (i.e., the model cannot be too complex) but also because most of our sample did not leave their homes on the survey reference day. In the context of the pandemic, objective variables like walkability are important but might not be as powerful after a year of varying lockdown conditions when older adults may have adjusted to a home-based lifestyle. Future work should include considerations of activity space and microenvironments, especially to understand how older adults are shedding pandemic habits.

## Conclusion

This study on older Chinese migrant populations during the second year of the COVID period contributes to the literature of older migrant loneliness and health in two important ways. First, this study reveals that access to public transit remained crucial for older migrants to maintain their community mobility even when they were experiencing travel restrictions, as evidenced by a positive relationship between transit density and community mobility. Second, we find that higher community mobility is associated with lower loneliness scores, and migrants with higher ICT use had better physical health scores even when controlling for exercise frequency. Taken together, these findings show that there is potential to improve older Chinese migrants’ loneliness and physical health through targeted built environment and technological interventions aimed at improving community mobility and ICT use behaviors. 

## Electronic supplementary material

Below is the link to the electronic supplementary material.

Supplementary Material 1

## Data Availability

The older Chinese migrant dataset generated and analyzed during the current study is not publicly available due privacy concerns but the data are available from the corresponding author on reasonable request. Walkability data are publicly available from Statistics Canada (https://www150.statcan.gc.ca/n1/pub/17-26-0002/172600022020001-eng.htm), GTFS data used to generate transit density are available from Transitfeed (https://transitfeeds.com/).

## Footnotes

1 Time in Canada was used as a control in a different iteration of the model but was not significant and worsened model fit.

## Declarations

### Authors’ information

The first author (AD) does not speak Mandarin Chinese, the language used to collect all data for this study. The survey instruments, consents, etc. were translated by BL, a native Mandarin speaker. XM and ZL, who are both native Mandarin speakers, commented on the final translation of materials.
