# Supplementary material for: Mobility, ICT, and health: a built environment investigation of older Chinese migrants’ social isolation and loneliness
Source: BMC Public Health. 2025 Feb 7;25:513. doi: 10.1186/s12889-025-21750-3 (PMC11806903; doi:10.1186/s12889-025-21750-3)
Supplement: Supplementary file 1 — Supplementary Material 1 [file 12889_2025_21750_MOESM1_ESM.docx]

## Appendices

**Appendix 1. Older Chinese migrant household members.**

| **Housemate** | **n (%) indicating affirmative** |
| --- | --- |
| Spouse | 74 (77.08) |
| Adult children | 27 (28.13) |
| Grandchildren | 13 (13.54) |
| Other relative | 3 (3.13) |
| Friend | 2 (2.08) |
| None (lives alone) | 14 (14.58) |

**Appendix 2. Map of older Chinese migrant sample.**

**
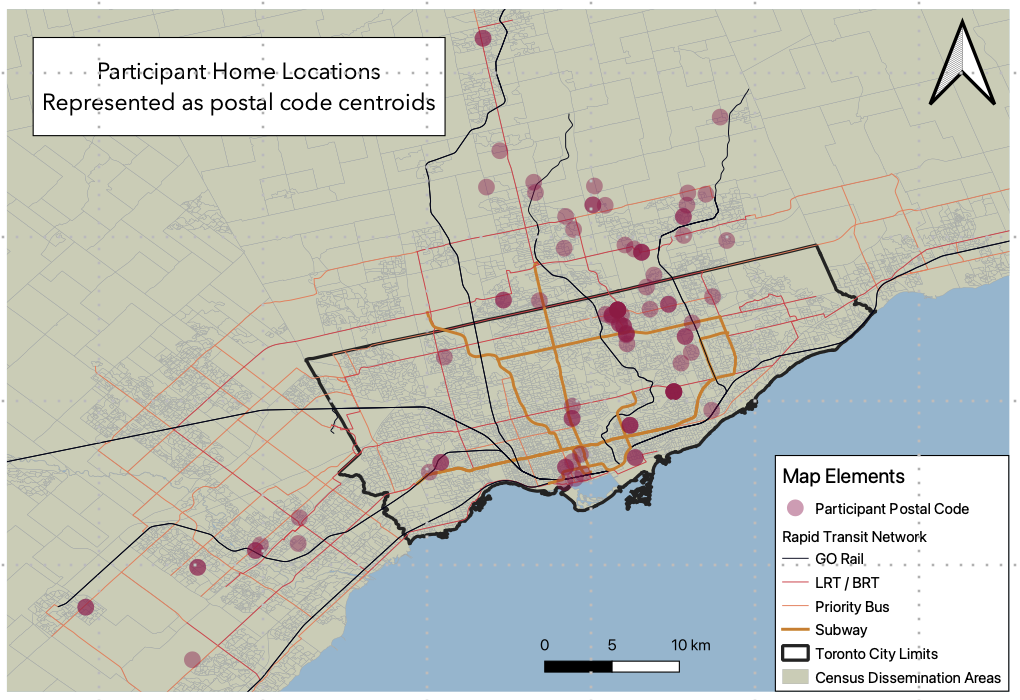
**

**Appendix 3. Chapter 4 Structural Equation Model fit statistics.**

| **Statistic** | **Observed value** | **Acceptable value** |
| --- | --- | --- |
| **Chi-square, baseline model** | 323.78 | - |
| **Chi-square, model** | 157.565* | - |
| **CFI** | 0.955 | > 0.95 |
| **TLI** | 0.972 | > 0.95 |
| **RMSEA** | 0.028 | < 0.08 |
| **RMSEA 90% CI** | [0.000, 0.057] | [0.000,0.999] |
| **WRMR** | 0.802 | < 1 |
| **p*-value > 0.05, which is desired for this statistic. | | |
